# Flux balance analysis-based metabolic modeling of microbial secondary metabolism: Current status and outlook

**DOI:** 10.1371/journal.pcbi.1011391

**Published:** 2023-08-24

**Authors:** Sizhe Qiu, Aidong Yang, Hong Zeng

**Affiliations:** 1 School of Food and Health, Beijing Technology and Business University, Bejing, China; 2 Department of Engineering Science, University of Oxford, Oxford, United Kingdom; CPERI, GREECE

## Abstract

In microorganisms, different from primary metabolism for cellular growth, secondary metabolism is for ecological interactions and stress responses and an important source of natural products widely used in various areas such as pharmaceutics and food additives. With advancements of sequencing technologies and bioinformatics tools, a large number of biosynthetic gene clusters of secondary metabolites have been discovered from microbial genomes. However, due to challenges from the difficulty of genome-scale pathway reconstruction and the limitation of conventional flux balance analysis (FBA) on secondary metabolism, the quantitative modeling of secondary metabolism is poorly established, in contrast to that of primary metabolism. This review first discusses current efforts on the reconstruction of secondary metabolic pathways in genome-scale metabolic models (GSMMs), as well as related FBA-based modeling techniques. Additionally, potential extensions of FBA are suggested to improve the prediction accuracy of secondary metabolite production. As this review posits, biosynthetic pathway reconstruction for various secondary metabolites will become automated and a modeling framework capturing secondary metabolism onset will enhance the predictive power. Expectedly, an improved FBA-based modeling workflow will facilitate quantitative study of secondary metabolism and in silico design of engineering strategies for natural product production.

## 1. Introduction

The cellular metabolism in microorganisms is the set of biochemical reactions to maintain life, and it can be divided into 2 branches, primary and secondary metabolisms: primary metabolism generates energy and synthesizes cellular biomass, while secondary metabolism mediates microorganisms’ adaptation to the living environment [1]. Secondary metabolites have been defined as metabolites that are unessential for growth and reproduction [2–5], but they usually have specialized functions and the biosynthesis of them is sometimes species specific. Though secondary metabolites have little contribution to cell growth from the simple mass balance point of view, they are ecologically and evolutionarily important as they often contribute to inter-species antagonistic or mutualistic interactions [6] and responses to environmental stresses [7–9].

As a rich source of valuable natural products, microbial secondary metabolites have been contributing to pharmaceutical, cosmetic, food, and agricultural industries since the discovery of penicillin [10], for example, cyclic lipopeptides derived from *Bacillus subtilis* used as antibacterial agents in both medicine and agriculture [11], lactic acid bacteria-derived exopolysaccharides (EPSs) used as food additives [12], and sacran produced by cyanobacteria for skincare [13]. Compared to plant-based processes, microbial bioproduction processes are more efficient and implementable, as the doubling time of a microbial cell is usually 20 to 60 min [14], much shorter than that of a plant cell, which typically takes days [15]. In addition, a microbial system is more controllable than a plant system, as the latter is more subject to fluctuating environmental factors [16]. There have been many successful applications to optimize the microbial production of secondary metabolites, e.g., through eliminating competing pathways, using genome shuffling to obtain a high-yield strain [17], or fine-tuning gene expression by promoter engineering [18]. Overall, microorganisms are ideal platforms to produce secondary metabolites, due to the richness of valuable natural products and inherent advantages as cell factories.

Data-driven computational modeling has become widely used in the quantitative study of the synthetic biology of secondary metabolism [19] due to the fast growing amount of multiomics data and computational resources [20,21]. Two most commonly used modeling methods of cellular metabolism are differential equation-based models [22,23] and flux balance analysis (FBA)-based models [24]. Admittedly, differential equation-based models can be easily connected with process control, but most of them treat the whole metabolic network as a “black box” [25,26]. On the other hand, fine-grained “white-box” differential equation-based models [23] are typically costly to construct due to limited data availability [19,27] and complex enzyme kinetic mechanisms [28]. Alternatively, the reconstruction of a genome-scale metabolic model (GSMM) is fast and mostly automated [29], and the major analytical approach of a GSMM, i.e., FBA using linear programming, is less computationally expensive than solving a large-scale differential equation set [30,31]. Therefore, this review focuses on FBA-based modeling techniques and GSMMs integrated with secondary metabolic pathways (smGSMMs).

In recent years, utilizing FBA to model and engineer secondary metabolism has gained significant interest in synthetic biology, especially in the production of antibiotics [32]. Consequently, review articles on this topic have emerged: Breitling and colleagues focused on modeling challenges caused by incomplete and uncertain information and suggested quantitative consideration of parameter uncertainties [19]; Mohite and colleagues provided an overview of various smGSMMs for actinomycetes’ and modeling techniques applied for the production of antibiotics [32]; Weber and Kim summarized tools for searching secondary metabolites’ biosynthetic gene clusters (BGCs) and smGSMM reconstruction [21]. Nevertheless, existing reviews cover either BGC identification which is only part of the modeling process or modeling techniques for a specific class of secondary metabolites; therefore, this review intends to elucidate this topic more holistically and generically via presenting the current progress of the complete modeling process, i.e., from building an smGSMM for target secondary metabolites to predicting secondary metabolite production flux within the scope of FBA-based modeling techniques. We begin with compiling recent developments on the reconstruction of secondary metabolic pathways, which are necessary for building smGSMMs. Then, we move on to discuss FBA-based modeling techniques for predicting secondary metabolite production, where various modeling techniques that manage to capture secondary metabolism are critically reviewed. Finally, suggestions are given to potential extensions of FBA to improve the prediction accuracy of secondary metabolite production. Unlike an early relevant work that discusses how to incorporate parameter uncertainties that result from incomplete information of secondary metabolism [19], this review directs attention to approaches that manage to quantitatively capture secondary metabolite production with pathway reconstruction and FBA-based modeling techniques developed in recent years.

## 2. Pathway reconstruction for microbial-derived secondary metabolites

**Fig 1:**
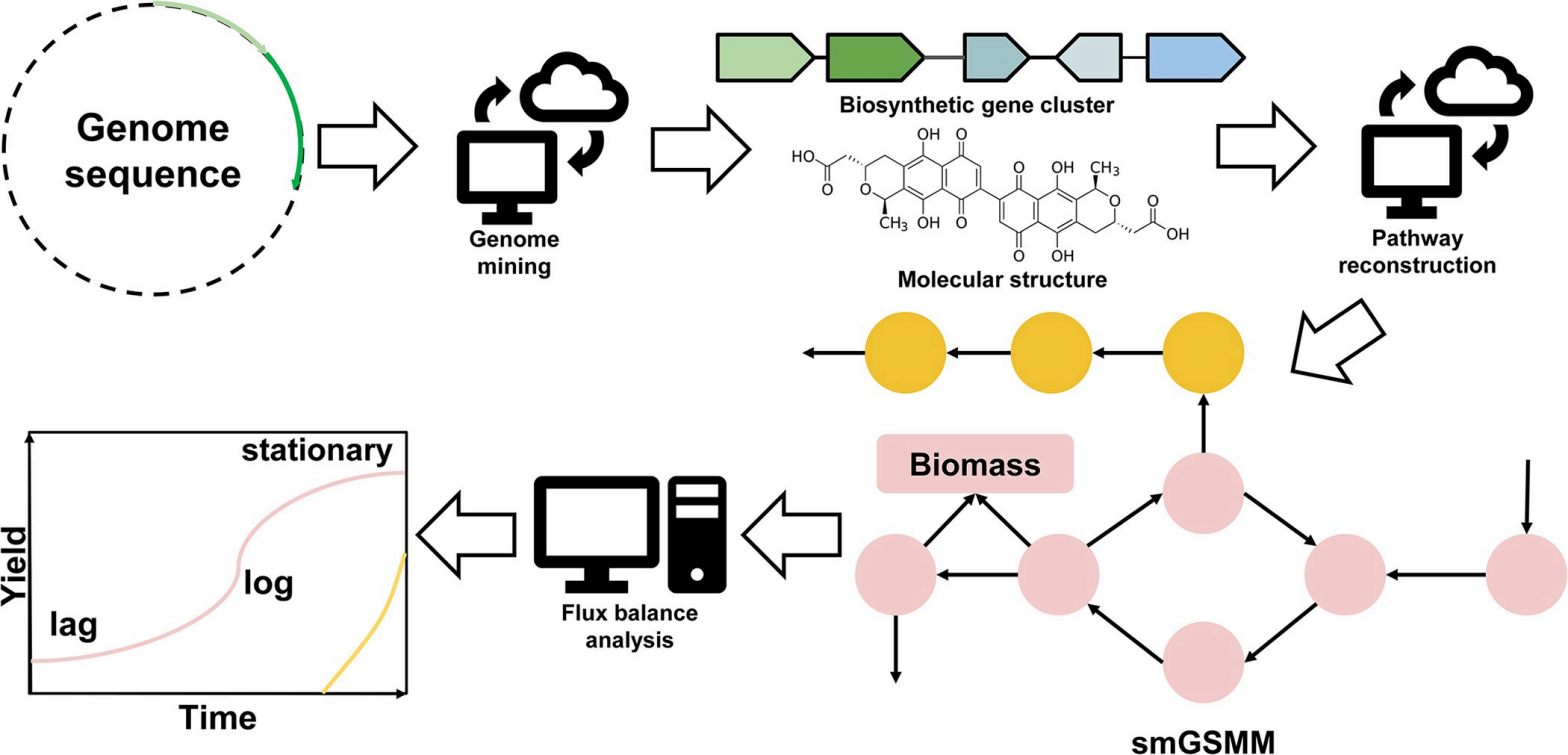
Graphical abstract of the workflow of FBA-based metabolic modeling for secondary metabolism. Orange circle: secondary metabolite; pink circle: primary metabolite. FBA, flux balance analysis; smGSMM, genome-scale metabolic model with secondary metabolic pathway.

To model secondary metabolism, 3 key steps are needed: (1) the identification of secondary metabolites and associated BGCs through genome mining; (2) reconstruction of secondary metabolic pathways; and (3) the simulation of metabolic fluxes (**Fig 1**). The first 2 steps are the basis of the third step. For BGC identification, a number of well-developed genome mining tools that can find BGCs for the biosynthesis of secondary metabolites are available, such as antiSMASH [33], PRISM [34], or BAGEL [35]. The development of genome mining tools in recent years has been reviewed by many well-written articles [21,36–41], from which interested readers can find in-depth information. Compared to genome mining tools applicable to finding BGCs of secondary metabolites, commonly used metabolic network reconstruction tools show limitations in assembling the biosynthetic pathways of secondary metabolites, yet there exists a few tools capable of addressing such a challenge [42,43].

### 2.1. Limitations of current genome-scale metabolic network reconstruction tools on assembling secondary metabolic pathways

The computational modeling of secondary metabolism requires in silico biosynthetic pathway reconstruction of secondary metabolites. Commonly used automated GSMM reconstruction tools are CarveMe [44], ModelSEED [45], RAVEN [46], Merlin [47], AutoKEGGRec [48], AuReMe [49], MetaDraft [50], Pathway Tools [51], FAME [52], and GEMSiRV [53]. They rely on metabolic reaction databases: BiGG [54], SEED [45], MetaCyC [55], and KEGG [56]. Among them, BiGG and SEED both integrate and standardize genome annotations, reactions, and GSMMs of different microorganisms, but leave many gaps in peripheral pathways associated with secondary metabolites [44]. MetaCyC contains 747 secondary metabolic pathways [57], but most of them are plant specific [58,59]. Reconstruction of 24 penicillium GSMMs by Prigent and colleagues reveals that MetaCyc allows automatic reconstruction of 34 biosynthetic pathways of terpenoids, polyketides (PKs), phenylpropanoids, alkaloids, and nonribosomal peptides (NRPs) [57]. Compared to MetaCyc, KEGG has significantly depleted “Metabolism Of Terpenoids And Polyketides” and enriched “Alkaloid And Other Secondary Metabolite Biosynthesis” pathway classes [60]. Despite advances in smGSMMs of penicillium [57] and actinomycetes [32], the incomplete description of species-specific secondary metabolism in databases still makes it hard to reconstruct secondary metabolic pathways based on genome annotation alone without supplementary experimental information [19,61]. In short, among the currently available genome-scale metabolic network reconstruction tools, it is difficult to recommend a state of the art one for reconstructing complete biosynthetic pathways of secondary metabolites.

Given the issues presented in automated pathway reconstruction tools, manual curation is sometimes used as an expedient solution, e.g., the paulomycin pathway in the smGSMM of *Streptomyces albus J1074* [62], the lumped biosynthetic reaction of EPS from monosaccharides in *Lactobacillus casei LC2W* [63]. However, manual curation is laborious, inefficient, and subject to human error. Most manual approaches are not fine-grained enough, which means intermediary metabolites are left out. As a result, smGSMMs are incapable of accounting for bottlenecks that affect secondary metabolite production, such as precursor depletion, poor enzyme capacity of intermediate step, or toxic intermediate accumulation [19,64]. The limited performance calls for pathway reconstruction tools specialized for secondary metabolism. Fortunately, there are still some tools for automated pathway reconstruction of certain secondary metabolites, which were developed via bottom-up (BGC-based) or top-down (retrosynthesis-based) approaches (**Table 1**).

**Table 1:** Automated pathway reconstruction tools for microbial secondary metabolism.

Tool	Scope	Input	Output	Reference
BiGMeC	PKs, NRPs	Genbank files of BGCs from antiSMASH	Json files containing reconstructed pathways	[42]
DDAP	Type I PK synthase	Fasta/genbank/csv files of polyketide synthase sequences	A list of possible pathways and the associated product SMILES strings	[65]
RetroPath 2.0	All classes of secondary metabolites	Source and sink compounds’ SMILES strings and reaction rules	A reaction network linking the source set to the sink set	[67]
BioNavi-NP	All classes of secondary metabolites	Products’ SMILES strings and reaction rules	Possible precursors and biosynthetic pathways	[68]

BGC, biosynthetic gene cluster; NRP, nonribosomal peptide; PK, polyketide.

### 2.2. BGC-based pathway reconstruction

Firstly, the bottom-up approach, namely the BGC-based approach, reconstructs secondary metabolic pathways from identified BGCs. Genome mining tools such as antiSMASH identify BGCs of secondary metabolites and output genbank files containing annotated functional domains. Subsequently, reconstruction tools such as **BiGMeC** [42] and **DDAP** [65] take identified BGCs as inputs and assemble reactions from template smGSMMs (e.g., Sco-GEM for *Streptomyces coelicolor* [66]) or pre-curated pathway databases (e.g., DDPA database for type I PKS pathways [65]) based on annotated genes in BGCs. The BGC-based method reconstructs genome-scale biosynthetic pathways of secondary metabolites with sufficient accuracy; however, its scope is limited, i.e., the reconstruction usually works for PKs and NRPs only [42], because genome mining tools like antiSMASH or PRISM sometimes cannot detect BGCs of other secondary metabolites, such as EPSs or fatty acid derivatives [40]. Also, the pre-curated databases and template smGSMMs have low coverage of biosynthetic pathways of secondary metabolites other than PKs or NRPs.

### 2.3. Retrosynthesis-based pathway reconstruction

Unlike reconstructing pathways from BGCs, a “top-down” approach predicts pathways based on retrosynthesis of end products. Tools like **RetroPath 2.0** [67] or **BioNavi-NP** [68] can predict reactions to produce target secondary metabolites using the retrosynthesis algorithm. This algorithm uses a dataset of curated reactions from databases, such as SimPheny [69] or BNICE [70], as the input to generate reaction rules, which are used to decompose the end product’s molecular structure into possible precursors [71]. The final output is a route linking precursors to the end product, in which all reactions are essential for their viability [67]. For example, BioNavi-NP decomposes Sterhirsutin J into potential candidate precursors, such as colletorin D acid, and those candidates’ biosynthesis are further traced back to simple building blocks (e.g., malonyl CoA), finally outputting most possible biosynthetic pathways [68]. The advantage of retrosynthesis is that the pathway reconstruction is no longer restricted to certain classes of secondary metabolites, as its pathway prediction is based on reaction rules and the molecular structure of the end product. However, retrosynthesis might give multiple solutions and the suggested solutions are not always consistent with the actual genome of the studied microbe, since reaction rules from databases are not species specific. Therefore, for better reconstruction accuracy, users need to adjust either the list of reaction rules or the reconstructed pathway based on the organism’s protein sequences, before making predictions.

In short, to build smGSMMs, the practical approach seems to be first reconstructing secondary metabolic pathways with automated methods, and then integrating them to the primary metabolic network. For PKs and NRPs, BGC-based tools (BiGMeC and DDAP) are advantageous, while retrosynthesis can predict the biosynthetic pathways for other secondary metabolites.

## 3. Towards predicting microbial production of secondary metabolites: FBA-based modeling techniques

### 3.1. Challenges faced by flux balance analysis to predict secondary metabolite production

FBA models cellular metabolism by utilizing linear programming to compute metabolic fluxes, optimizing an objective function, usually biomass formation (*v*_*growth*_) or a tailored objective (*c*_1×R_*[*v*_1_, *v*_2_,…,*v*_*R*_]^*T*^, *c*_1×*R*_ is a vector of coefficients, T stands for matrix transpose) (Eq 1), *v* stands for the reaction flux. The linear programming is solved in a constrained solution space of mass conservation (Eq 2) and upper/lower bounds of reaction fluxes, *v*_*i*,*max*_ and *v*_*i*,*min*_ (Eq 3) [24]. Eq 2 is based on the pseudo-steady state assumption, and hence, FBA is often applicable only for modeling different stabilized steady states but not transient phases where intracellular metabolite concentrations will change [72].


$$Maximize\;objective=v_{growth}\;or\;c_{1\times R}*[v_{1},v_{2},\ldots ,v_{R}{]}^{T}$$ (1)

$${Mass\;conservation:\;S}_{M\times R}*[v_{1},v_{2},\ldots ,v_{R}{]}^{T}=0$$ (2)

$${Upper/lower\;bounds:\;v}_{i,min}{\leq v}_{i}\leq v_{i,max}$$ (3)

*v*_*growth*_ is the biomass formation rate normalized to 1 gram dry weight, also considered as the growth rate. *S*_*M*×*R*_ is the stoichiometric matrix of the metabolic network with *M* metabolites and *R* reactions. A lot of toolboxes have been developed for conducting FBA, such as COBRA Toolbox [73], MetaFlux [74], and FBA-SimVis [75], and it has become increasingly common to use FBA to quantitatively study cellular metabolism, e.g., elucidation of metabolic responses to different culture conditions [76,77], identification of gene knockout to improve metabolite production [78], prediction of metabolite cross-feeding in microbial communities [79].

Although FBA has been successful in predicting microbial growth and primary metabolism, its application in predicting secondary metabolite production faces challenges from several different aspects. The conventional FBA is tailored to simulate the growth and metabolism of microbes in the exponential phase, which is not ideal for simulating secondary metabolites’ biosynthesis that typically occurs in the stationary phase or late growth phase [32,72]. Ergo, the first apparent challenge is the unsuitable objective function. Generally, the maximization of biomass synthesis is used as the objective function, based on the assumption that cells use the available nutrients most efficiently for biomass synthesis [80]. Consequently, the metabolic fluxes involved in the biosynthesis of secondary metabolites, which are not essential components of biomass, are often neglected. Therefore, selecting a suitable objective function is crucial when simulating the metabolic switch from primary metabolism to secondary metabolism using FBA [32]. Another inherent shortcoming of FBA is its lack of characterization of regulation. The metabolic switch is controlled by gene expression regulation, and therefore, the prediction of secondary metabolite production will become more accurate if FBA incorporates the gene regulatory network (GRN) [81]. Besides, the overlook of environmental stresses (such as temperature [7,82], pH [83], nutrient availability [84,85], etc.), which impact the expression of enzymes in secondary metabolic pathways, also limits the accuracy of predictions. The optimality principle used to predict microbial metabolism should keep the balance between biomass formation and minimization of death under stress conditions, but the latter is ignored in conventional FBA [72]. Without incorporating important cellular activities such as stress response, FBA would not be able to predict nongrowth associated metabolic fluxes.

### 3.2. Existing FBA-based modeling techniques for secondary metabolism

To address the limitations of conventional FBA on secondary metabolism discussed in Section **3.1**, several techniques have been developed, including: (1) adding the targeted secondary metabolite into the growth function (biomass formation); (2) switching from the classical biomass formation objective to a secondary metabolism-associated objective; (3) estimating metabolic fluxes through sampling in a strictly constrained space; and (4) integrating gene expression data in FBA. The first 3 techniques aim to overcome the limitation caused by the unsuitable objective function, while the last one manages to incorporate gene expression regulation. The existing modeling techniques and their applications are summarized in **Table 2**, and their predictive power is also discussed.

**Table 2:** Summary of different modeling techniques to predict secondary metabolite production.

Secondary metabolite	Organism	Modeling technique	Predictive power	Reference
ACT, RED	*Streptomyces coelicolor*	Add the secondary metabolite into biomass and maximize biomass formation	Good accuracy but restricted by settings	[87,88]
Clavulanic acid	*Streptomyces clavuligerus*	Use ATP yield as the objective function	Good accuracy and unrestricted by settings	[89]
Riboflavin	*Ashbya gossypii*	Use secondary metabolite yield as the objective function	Good accuracy and unrestricted by settings	[90]
Acetoin, diacetyl, acetaldehyde, benzaldehyde, 2,3-butanediol, and amino acid-derived flavor metabolites, 2-methylbutanal, 2-methylpropanoic acid, etc.	*Lactococcus lactis subsp*. *cremoris*, *Lactococcus lactis subsp*. *lactis*, *Streptococcus thermophilus*, *Leuconostoc mesenteroides*	Maximize secondary metabolite yield in the solution space of FVA	Not quantitatively examined	[92]
ACT, RED, CDA, Cpk	*Streptomyces coelicolor*	Random sampling with enzyme constraints	Qualitatively accurate but not quantitatively examined	[66]
ACT, RED	*Streptomyces coelicolor*	Integration of transcriptomics data into FBA	Low quantitative accuracy	[97]

ACT, actinorhodin; CDA, calcium dependent antibiotics; Cpk, coelimycin P1; FBA, flux balance analysis; FVA, flux variability analysis; RED, undecylprodigiosin.

**Fig 2:**
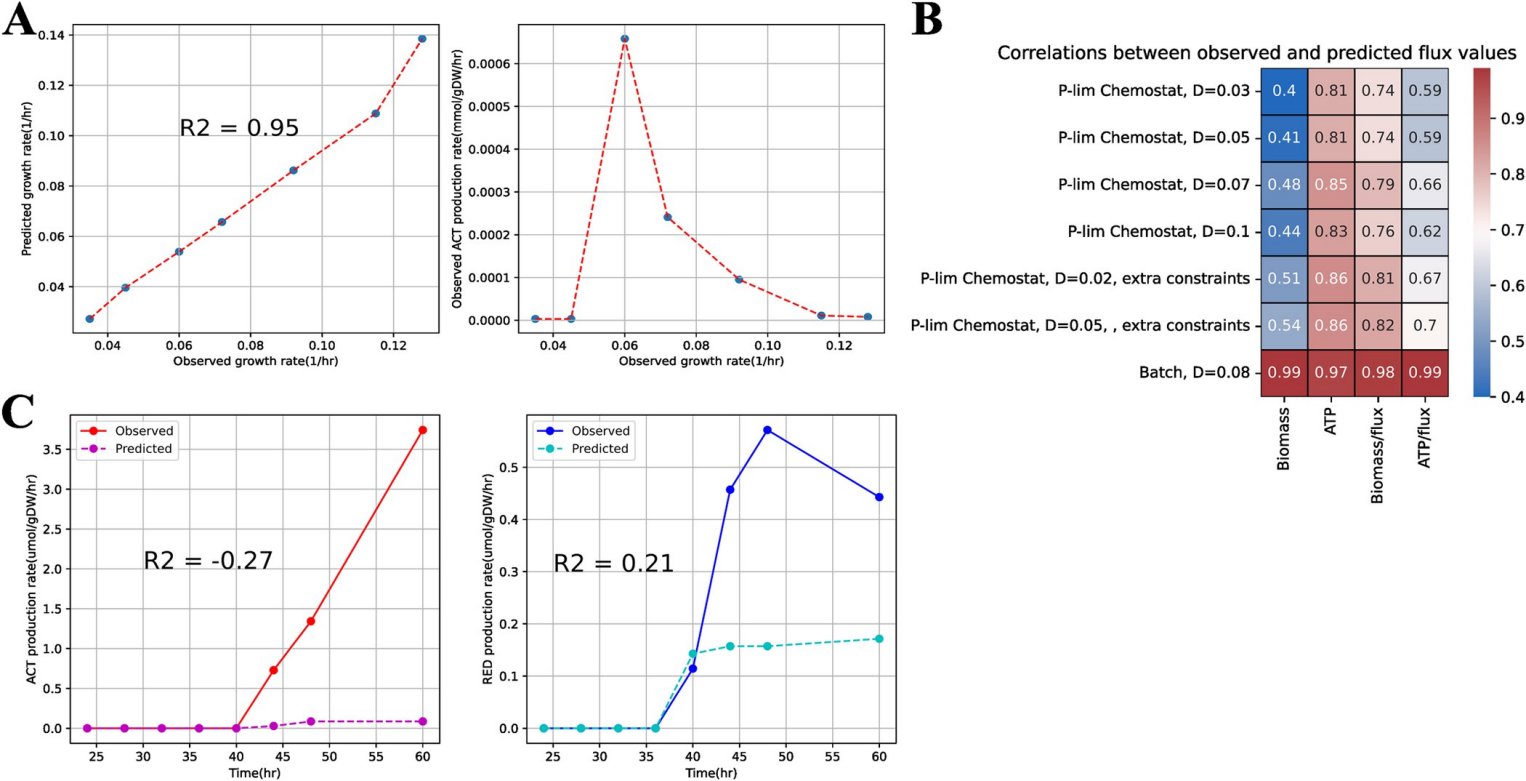
Quantitative assessment of existing FBA-based modeling techniques for predicting secondary metabolite production. (A) Comparison of predicted and observed growth rates and ACT production fluxes at different observed growth rates in Alam and colleagues [87]. (B) Comparison of 4 different objective functions used in FBA to predict both primary metabolism and clavulanic acid production flux [89]. Correlation scores are computed for predicted and observed fluxes. P-lim: limited phosphorus content. (C) Comparison of predicted and observed production fluxes of ACT and RED in Kim and colleagues [97]. ACT, actinorhodin; FBA, flux balance analysis.

Firstly, the flux distribution obtained from conventional FBA highly depends on the specific objective function used. If maximizing biomass formation is selected as the objective function, as commonly done, FBA would predict zero flux through the secondary metabolic pathways as it does not contribute to cell growth [86]. To resolve this type of limitation, it has been proposed to add the secondary metabolite into the biomass formation reaction, so that FBA will optimize both the synthesis of biomass precursors and the target secondary metabolite. This modeling technique was used to simulate the antibiotic production in *Streptomyces coelicolor*: based on the observed antibiotics production rate, the amount of antibiotics was increased dynamically when the cell transitioned from primary metabolism to secondary metabolism [87,88]. Because stoichiometric coefficients of undecylprodigiosin (RED) and actinorhodin (ACT) in biomass formation objective function were set based on experimental measurement, the accuracy in predicting the growth rate, R-squared value = 0.95, could reflect the accuracy of predicting secondary metabolite production (**Fig 2A**). This technique could account for the metabolic switch, as the studies by Alam and colleagues [87] and Amara and colleagues [88] demonstrated significant correlations between gene expression levels and predicted metabolic fluxes for most genes in secondary metabolic pathways. However, anti-correlations of predicted fluxes and gene expression levels for some genes in calcium-dependent antibiotics (CDAs) and RED biosynthesis indicated that the failure of including regulatory constraints was an important source of error [87,88]. From the perspective of modeling technique, in this case, FBA still optimizes the growth rate and makes the production of secondary metabolites growth-associated, which contradicts with the biological fact that the biosynthesis of most secondary metabolites is growth-unassociated. Also, this modeling technique manually fixes the ratio between secondary metabolite production and biomass formation (i.e., product yield per unit biomass) with experimental measurements, which restricts its applicability for cases where the yield of the secondary metabolite is varied or unknown. Due to the lack of a solid biological basis, this approach can only model flux distributions if the microbial cell behaves according to the artificially adjusted objective function. Though such an approach can, to some extent, reflect statuses of primary and secondary metabolisms, it can hardly be used directly for design purposes, because it cannot predict secondary metabolite production for different design settings of interest (e.g., different growth media, different strains).

As an alternative to the manipulation of the biomass formation while still adopting the classic objective function that maximizes biomass growth, switching to a new objective function can avoid introducing inappropriate growth association. The commonly used candidates of alternative objective functions are ATP yield, ATP yield per unit flux, biomass formation per unit flux, and target secondary metabolite production. Toro and colleagues compared 4 different objective functions’ performances on both primary metabolism and clavulanic acid production of *Streptomyces clavuligerus* [89], and the study found that maximization of ATP yield could predict primary metabolism and clavulanic acid production flux with best accuracy (**Fig 2B**). ATP yield maximization can be assumed to be the objective for microorganisms in both exponential growth and stationary phases, as the cell is always in need of energy to maintain its activity. A more direct objective function is to maximize the target secondary metabolite production. In the simulation of riboflavin overproduction by *Ashbya gossypii*, Ledesma-Amaro and colleagues assumed that *Ashbya gossypii* in the stationary phase switched from maximizing biomass formation entirely to maximizing riboflavin production [90]. The result showed that the simulated value of riboflavin production rate, 0.0156 mmol/gDW/h [90], was close to the experimental value, 0.0126 mmol/gDW/h [91]. Using flux variability analysis (FVA) to maximize the target secondary metabolite production from the flux solutions that maximize biomass growth is another approach, which has been used in modeling the production of flavor metabolites, such as acetoin, in lactic acid bacteria [92], but this approach has not been quantitatively examined with experimental data. The mathematical formulation of secondary metabolism-associated objective function in place of biomass formation has shown its ability to predict the production of some secondary metabolites, independently of condition-specific experimental data. However, changing the objective function may be too simplistic to account for metabolic activities that keep the balance of growth and minimization of death (stress resistance) as discussed in Section 3.1.

Since the FBA solution can be inaccurate with an unsuitable objective function, a modeling technique that is independent of objective functions has been adopted to resolve the issue. In the simulation using the Sco-GEM model, a consensus smGSMM for *Streptomyces coelicolor*, the metabolic fluxes were strictly constrained with the enzyme capacity, *v*≤*k*_*cat*_[*E*]. *k*_*cat*_ is the turnover rate and [*E*] is the concentration of the enzyme. Fluxes were approximated using unbiased random sampling in the constrained solution space instead of optimizing a defined objective function [66]. The metabolic switch from glycolysis, fatty acid, and nucleotide biosynthesis to ACT, RED, CDA, and coelimycin P1 (Cpk) biosynthesis was captured by this technique [66], but no comparison of predicted and observed reaction fluxes was performed for quantitative validation. Because random sampling without a highly constrained space will output multiple flux states with wide ranges, the accuracy of the predicted fluxes relies on the strict constraints imposed by quantified enzyme protein concentrations and enzyme kinetic parameters, which are strain specific and condition specific. Therefore, this modeling technique cannot compute metabolic fluxes through secondary metabolic pathways without proteomic data measured under a given setting. The cost of proteomics quantification makes it hard to predict secondary metabolite production with different growth conditions.

Modeling techniques discussed above do not take into account the inherent lack of characterizing regulation in conventional FBA. Reactions in a GSMM, if not gap filled, have associated gene-protein-reaction (GPR) rules, allowing the integration of quantitative gene expression data, such as transcriptomics or proteomics. If gene expression levels are integrated into FBA, the predicted fluxes can reflect metabolic enzyme activities and thus characterize regulations. The generic algorithm is to minimize the utilization of reactions with low gene expression levels and allow reactions with high gene expression levels to have higher absolute flux values.

There have been various developed techniques for integrating gene expression data into FBA, e.g., GIMME [93], E-flux [94], iMAT [95], but most were applied only on primary metabolism [96]. However, there exists a transcriptomics-based strain optimization tool for secondary metabolite production that uses iMAT to predict the secondary metabolism of *Streptomyces coelicolor* [97]. The comparison of different gene expression integrated FBA modeling techniques, iMAT was found to be the only technique that can capture the onset of secondary metabolism in *Streptomyces coelicolor* [97]. Nonetheless, the discrepancy between predicted and observed production fluxes of ACT and RED indicates that iMAT only performs well in a qualitative manner (**Fig 2C**). Also, this technique relies on condition-specific gene expression data, limiting the scope of extrapolation. It can predict metabolism for various design settings of interest only if different gene expression data corresponding to the different settings are provided. Otherwise, this technique, to a large extent, can merely compute the flux distribution corresponding to a specific set of gene expression levels. In short, to our knowledge, so far there has not been a gene expression integrated modeling technique that predicts secondary metabolite production with quantitative accuracy.

In a nutshell, existing FBA-based modeling techniques that manipulate the objective function cannot capture 2 distinct life objectives of a microbial cell concurrently, i.e., maximizing growth and minimizing death [72]. Besides, how to formulate a mathematical model that keeps the balance between the 2 objectives still remains an open question. Furthermore, no mechanistic representation of gene expression regulation upstream to metabolism has been built and integrated in FBA to make better use of omics datasets than simply determining “on” and “off” of reactions for fixed settings. In summary, for existing FBA-based modeling techniques that predict secondary metabolite production, those without integration of gene expression data are either limited to specific cases or too simplistic that only work under certain ideal assumptions, while gene expression integrated techniques are scarce and unable to make predictions for different settings without costly data generation.

### 3.3. Potential extensions of FBA towards predicting secondary metabolite production

The associated GPR rules, already targeted by some of the existing approaches to predict the secondary metabolite production as summarized in **Section 3.2,** suggests that the ability to determine the activity of enzymes in secondary metabolic pathways could be the key to the accuracy of modeling the secondary metabolite production. Here, this review proposes 2 potential extensions of FBA that may resolve issues of existing modeling techniques discussed in Section 3.2.

### 3.3.1. Constrained proteome allocation for secondary metabolism

Proteome allocation, namely the distribution of proteome resources in different pathways, governs cellular metabolism by controlling maximum reaction fluxes [98]. The constrained proteome allocation theory has been proposed to model the competition for proteome resources among different functional sectors such as catabolism, anabolism, transportation, etc., characterizing the coordination of proteome partitions and cellular metabolism under different growth conditions [99–101]. The theoretical model has been applied in various FBA-based models, such as CAFBA [102] or ME-model [103], via converting it into proteomic constraints on reaction fluxes. There are different ways to divide the proteome based on the modeling requirement. For example, the proteome was divided into fermentation, respiration, and biomass formation sectors in the prediction of overflow metabolism in *Escherichia coli* [104]. Usually, glycolytic enzymes are clustered as the sector of catabolism or energy, membrane transporter proteins are clustered as the sector of transportation, and a lumped proteome resource for the growth function (biomass formation) is considered the sector of anabolism [102,105,106]. The neglect of other enzymes’ proteome costs normally will not significantly affect the simulation, as fluxes through those pathways are far smaller than that through central carbon metabolism [102,105,106].

**Fig 3:**
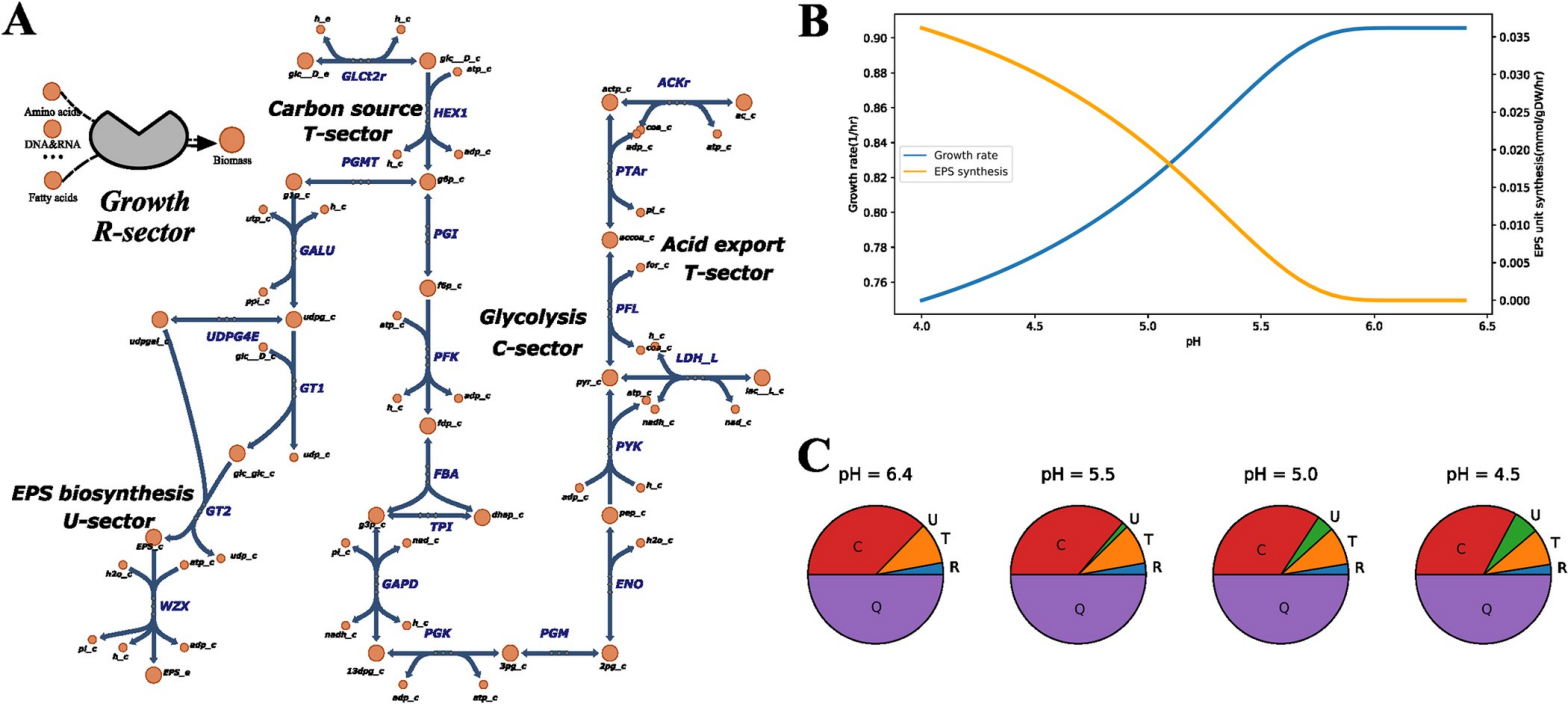
An illustrative example of pH-induced EPS production in lactic acid bacteria, used to explain the constrained proteome allocation model for both primary and secondary metabolism. (A) The metabolic network of lactic acid bacteria for both primary metabolism (C, R, T sectors) and secondary metabolism (U sector). (B) Simulated metabolic response to pH: the increase of acidity inhibits the growth rate and induces EPS production. (C) Simulated proteome allocation in response to pH: the increase of acidity activates secondary metabolism, and more proteome resources get allocated to the U sector. Note: this “toy” model is for illustration only. EPS, exopolysaccharide.

Though constrained proteome allocation-embedded FBA models have achieved good accuracy in case studies such as predicting overflow metabolism in *E*. *coli* [105] or explaining lactic acid production in lactic acid bacteria [106], to our knowledge it has not been used to predict the secondary metabolite production yet. The proteome partitioning has not included a sector defined for secondary metabolism, as we found in various proteome allocation models. To apply proteome allocation to predict secondary metabolite production, one potential approach is integrating synthetic chemostat model (SCM) into FBA [72]. SCM is a differential equation-based model of microbial growth kinetics that divides cellular metabolism into a P-component for growth and a U-component for stress resistance [107]. Being a macroscopic bioreactor-level model, SCM is too coarse to characterize different functional proteome sectors, but the embedded concept can be used to modify the original constrained proteome allocation framework. If a U-sector containing enzymes in biosynthetic pathways of secondary metabolites is included and stress response is introduced into proteome allocation, then the modified model might be able to characterize both growth and stress response via proteome allocation at the metabolic switch that leads to secondary metabolite production. Below, an illustrative example of pH-induced EPS production in lactic acid bacteria [108,109] is presented for the proposed constrained proteome allocation model for both branches of metabolism (**Fig 3**).


$$\phi_{Q}(50\%)+\phi_{U}+\phi_{C}+\phi_{R}+\phi_{T}\leq 1$$ (4)

$$\frac{\phi_{U}}{\phi_{U}+\phi_{C}+\phi_{R}+\phi_{T}}\geq {k_{1}e}^{\frac{-k_{2}}{(6.5-pH{)}^{2}}},k_{1},k_{2}>0$$ (5)

$$v_{i}\leq kcat_{i}[E_{i}],\sum_{i}\left[E_{i}\right]\leq \phi_{x}{[P}_{TOT}],x=U,C,R,T$$ (6)

The cellular proteome is divided into inflexible housekeeping sector (Q-sector), catabolic sector (C-sector), ribosomal sector for protein translation (R-sector), transportation sector (T-sector), and secondary metabolism sector (U-sector). *ϕ*_*x*_ is the mass fraction of the sector *x*, and *ϕ*_*Q*_ is assumed to 50% [102,105,106] (Eqs 4, 6). The total amount of proteome resources is conserved (Eq 4). In response to environmental stress, which is acidity in this case, the fraction of proteome resources allocated to the U-sector, which is EPS biosynthesis, rises up (Eq 5). *k*_1_ and *k*_2_ in Eq 5 are empirical coefficients. The metabolic fluxes are constrained by the total amount of proteome resources allocated to the sector (Eq 6). [*E*_*i*_], *kcat*_*i*_ are the concentration and turnover rate of the enzyme *i*, respectively, and [*P*_*TOT*_] is the concentration of total cellular proteins. The proteome allocation of secondary metabolism (Eq 5) can materialize in different forms in actual implementation, e.g., modeling the consequence of differing proteomic costs caused for different stress factors, such as temperature, inhibitors, or nutrient limitation [102,110,111].

### 3.3.2. Combine gene regulatory and metabolic networks to capture secondary metabolism onset

**Fig 4:**
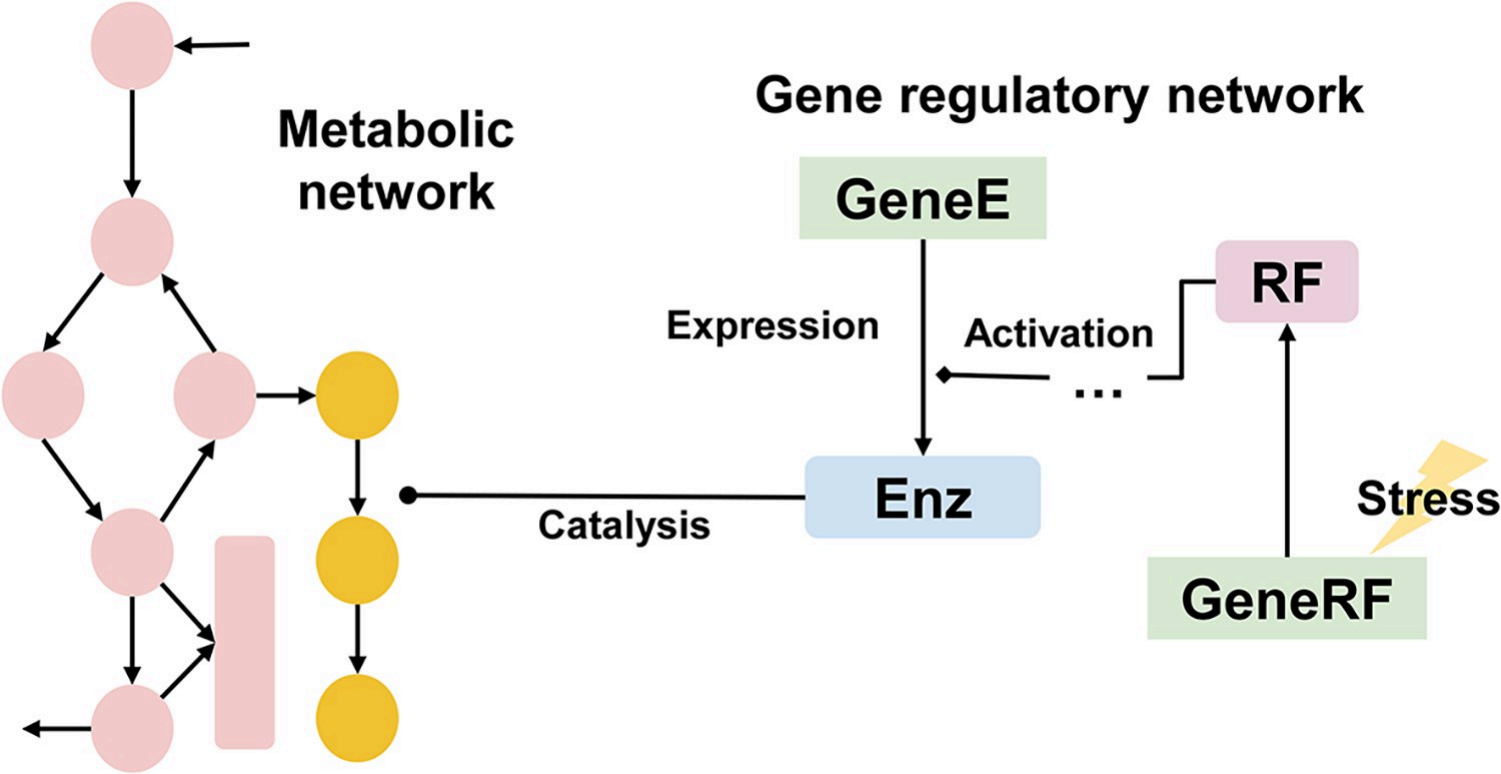
Schematic diagram of the combination of metabolic and GRNs to predict the secondary metabolite production. The stress signal stimulates the expression of RF, resulting in the activation of enzymes catalyzing reactions for secondary metabolite biosynthesis. Orange circle: secondary metabolite; pink circle: primary metabolite. Enz, enzyme; GRN, gene regulatory network; RF, regulatory factor.

Constrained proteome allocation-embedded FBA models can capture the shift in metabolic states via modeling resource distribution among functional proteome sectors [102]. However, they do not characterize regulatory interactions between regulatory factors (RFs) and genes at transcriptional or translational level. On the other hand, existing gene expression integrated FBA modeling techniques, such as iMAT, do not have a mechanistic representation of gene expression regulation, and hence, their applications on the prediction of secondary metabolite production are restricted by condition-specific data availability. Therefore, a regulatory network model needs to be combined with FBA, as illustrated in **Fig 4**, to resolve the limitation of direct integration of gene expression into FBA. Though not for predicting secondary metabolite production, several integrated regulatory-metabolic models, also called regulatory FBA (rFBA), have been reported for a while [112–115]. The generic framework of dynamic rFBA is a combination of FBA for intracellular metabolic fluxes, a Boolean logic network modeling regulatory interactions, and a set of differential equations modeling concentrations of RFs, biomass, and extracellular metabolites [116]. The rFBA model constrains metabolic fluxes by predicting the expression status of the gene associated with the reaction, which means that metabolic fluxes only flow through active reactions.

Enlightened by the idea of dynamic rFBA, if regulatory interactions related to secondary metabolism are elucidated in a Boolean logic network, dynamic simulation of gene expression regulation at metabolic switch can be performed, and thus quantitatively capture the onset of secondary metabolite production (**Fig 4**). The stress response can be appended into rFBA, as the process from stress sensing to regulation can be represented by signal transduction in a network [117], though such approach has not been applied to the modeling of secondary metabolism yet. In essence, rFBA has the potential to overcome the limitation of conventional FBA by including regulation, an important cellular activity apart from biomass formation, which will enhance the predictive power of FBA on secondary metabolite production.

## 4. Conclusion and outlook

This review summarized the current key challenges that limit the modeling of secondary metabolism, which are mainly derived from 2 aspects: (1) the difficulty in reconstructing the complete biosynthetic route of a secondary metabolite in an enzymatically detailed manner; and (20) the inability of FBA-based modeling techniques to make quantitatively accurate predictions for secondary metabolism under different conditions for engineering and practical purposes. As for the first challenge, except for PKs/NRPs, the pathway reconstruction for secondary metabolites generally lacks template models and databases containing information of biochemical reactions associated with secondary metabolism. Consequently, this process is much less automated. For example, although databases related with EPS biosynthesis are available, e.g., EPS-DB [118], CAZy [119], the information on enzymes and molecular structures is not assembled together, which makes automated reconstruction of EPS biosynthetic pathways currently infeasible.

The existing FBA-based modeling techniques that are intended to tackle the second challenge have been evaluated in this review. It shows that they are either too simplistic or have restricted application scopes. None of them are predictive enough to capture the production of secondary metabolites. Nevertheless, those modeling techniques show usefulness in certain cases, such as manipulating the objective function and incorporating transcriptomics data. With the aim of building a modeling technique that captures the mechanistic understanding of secondary metabolism, we have suggested the application of 2 potential extensions of FBA-based modeling techniques. They respectively incorporate constrained proteome allocation that explicitly account for the enzyme capacity for secondary metabolism and connect with the regulatory network model for gene expression states that control reaction activities with high resolution. Detailed implementation approaches in those directions remain to be explored, which we envisage will benefit from further and more mechanistic understanding of cellular responses leading to the production of secondary metabolites. Additionally, future curation works of proteomic and gene regulatory information, both in connection with typical or specific secondary metabolic pathways, will contribute to the advancement of those approaches.

When a high coverage of biosynthetic pathways for secondary metabolites is achieved in metabolic reaction databases for smGSMM reconstruction, and a modeling technique that accurately captures the mechanism of secondary metabolism is developed, the complex bioprocess of secondary metabolite production will then become more “white-boxed.” Researchers, particularly those in the field of synthetic biology, will be able to conduct more reliable in silico analysis of secondary metabolite production. This advancement will contribute to the development of model-based design and pathway engineering approaches aimed at controlling secondary metabolism, such as enhancing the productivity of high-value secondary metabolites.
